# A haplotype-resolved genome assembly and gene expression map of Cushion willow

**DOI:** 10.1038/s41597-025-05132-3

**Published:** 2025-05-13

**Authors:** Jindan Wang, Kaiyun Chen, Rengang Zhang, Yuan Huang, Jiahui Chen

**Affiliations:** 1 CAS Key Laboratory for Plant Diversity and Biogeography of East Asia, Kunming Institute of Botany, Chinese Academy of Sciences, Kunming, 650201 Yunnan P. R. China; 2 University of Chinese Academy of Sciences, Beijing, 100049 P. R. China; 3 School of Life Sciences, Yunnan Normal University, Kunming, 650092 Yunnan P. R. China

**Keywords:** Plant evolution, Natural variation in plants

## Abstract

*Salix brachista*, commonly known as Cushion willow, is a common component of subnival alpine assemblages and a dioecious or monoecious plant with a creeping stem and numerous lateral branches. Cushion willow takes cuttings more easier and has a specific sex system, making it a suitable system for studying the evolution of plant sex determination, adaptive evolution of alpine plants, and mining stress resistance gene resource that cope with the hostile alpine environment. Therefore, Cushion willow has potential value in genetic improvements for willows used as bioenergy crops, in gardening, and as ornamental plants. However, the genome of Cushion willow still contains some un-assembled repetitive sequences, and there is limited availability of a gene expression atlas, which hinders its potential use for the aforementioned purposes. Here, we updated the genome of Cushion willow to be haplotype-resolved and near telomere-to-telomere, and obtained a high-quality transcriptomic map. Our research provides a potential model species for alpine adaptive research, sex determination evolution studies, and improving willow crops.

## Background & Summary

*Salix* L. (commonly known as willows) is the largest genus of woody plants in the Northern Hemisphere within the family Salicaceae s.str., comprising ca. 400–520 species^1^. Willows have significant economic value due to their use in ornamental use, landscaping, soil engineering, wind prevention, etc^1–3^. Shrub willows have been identified as a promising biomass crop and are widely used for biomass production due to their ease of propagation and ability to grow quickly in short rotation coppice cycles with minimal fertilizer inputs. Therefore, they are considered the most suitable woody bioenergy crops and are widely planted^4^. To fully utilize the potential of renewable energy, it is important to maintain willows free of pests and diseases while improving yields without significantly increasing the need for fertilizers and water^5^. Willows are dioecious, though some species are monoecious, making them an excellent taxon for studying plant sex determination evolution in recent years. Willows exhibit both ZW and XY sex systems, and the location of their sex-determining regions is dynamic and varies among species^6–10^. Furthermore, willows are capable of vegetative reproduction from stumps, branches or roots, which allows for the simple generation of clones with the same genetic material^2^. These features make them convenient for control experiments aimed at studying gene functions.

Cushion willow (*Salix brachista* C. K. Schneider) is a common component of alpine subnival assemblages of the genus *Salix* L. It is a cushion plant with a creeping stem and a large number of lateral branches, growing to a height of usually no more than 5 cm^10^. Although mostly dioecious, we observed hermaphrodite flowers in one population, indicating that its sex determination region may be undergoing dynamic evolution. This makes it a very suitable system for studying willow sex determination evolution. It is mainly distributed in subnival zones with an elevation of around 4000 m (and occasionally found in lower elevations around 3000 m) in the Hengduan Mountains and adjacent areas, i.e. the eastern Himalaya and middle Yunnan Plateau. These high-altitude areas, such as the alpine subnival zone, are characterized by harsh environments, including strong solar radiation, strong winds, low temperatures, dramatic daily temperature fluctuations, hypoxia, poor soil, and uneven humidity and precipitation^11^. Plants in the alpine subnival zone, such as the Cushion willow, must cope with the harsh alpine environment. As a result, they have accumulated numerous stress resistance genes in their genome during their adaptive evolution to the alpine environment^12–14^.

In summary, Cushion willow is a suitable system for studying the evolution of plant sex determination, adaptive evolution of alpine plants, mining stress resistance genes in alpine plants, and studying related gene functions. Therefore, this species could have potential value in genetic improvements for willows used as bioenergy crops, in gardening, and as ornamental plants. However, the previously reported genome of Cushion willow still contains several repetitive sequences, such as telomeres and 5S rDNA, which remain un-assembled. Furthermore, there is a limited availability of a gene expression atlas. These factors hinder its potential value as mentioned above.

In this study, we present a high-quality genome and a transcriptome map of the Cushion willow, resolved by haplotype. (a) The haplotype-resolved, chromosome level genome was assembled using PacBio Revio System in circular consensus sequence, Illumina high-throughput chromosome conformation capture sequencing (Hi-C), Illumina high-throughput RNA-seq, and Nanopore full-length transcript technologies. We assembled 38 chromosomes that were classified as haplotype A and haplotype B. This result is consistent with previous karyotyping results (2n = 38)^15^. Haplotype A has a genome size of 401.5 Mb and contig N50 length of 22.6 Mb, while haplotype B has a genome size of 386.2 Mb and contig N50 length of 21.8 Mb (Table 1). The high-resolution genome annotated to 57,169 genes that contained 53,238 protein-coding genes and 3,931 RNAs (Supplement Table 5) (b) Transcripts were obtained from seven different organs, and full-length transcripts were obtained for mixed samples of these tissues. A total of 28,587 non-redundant transcripts were obtained from seven organs using Illumina sequencing. Additionally, 164.5 million full-length transcripts were obtained through Nanopore sequencing, with a mean read length of 981 bp and an N50 length of 1,194 bp. The Kyoto Encyclopedia of Genes and Genomes (KEGG) and Gene Ontology (GO) databases were used to annotate these transcripts and determine their function. Additionally, differentially expressed genes (DEGs) were identified in pairs of organs, and 33,414 alternative splicing (AS) events and 36,634 alternative polyadenylation (APA) sites were detected using full-length transcripts, which are more accurate than Illumina data (Figs. 5 and 6). The genome and transcriptome map of Cushion willow, resolved by haplotype, will provide valuable reference material for studying genetic improvement of Cushion willow plants and other alpine *Salix* species, as well as exploring the adaptive evolution of alpine extreme environments on the Qinghai-Tibet Plateau.

**Table 1:** Summary of the *S. brachista* genome assembly data.

Statistic	Haplotype A	Haplotype B
Total size (bp)	401,494,730	386,222,989
Number of gaps	1	1
Size of gaps (bp)	100	100
GC content (%)	34.88	34.88
Characteristic	Scaffold	Scaffold
Number of chromosomes	19	19
Max. (bp)	36,017,666	34,689,865
Mean (bp)	21,131,302	20,327,526
Min. (bp)	11,424,557	11,622,248
N10 (bp)	25,909,808	24,071,074
N50 (bp)	22,629,168	21,845,516
N90 (bp)	15,550,447	14,821,491
L10	2	2
L50	8	8
L90	16	17

## Methods

### Sample collection

For the extraction of genomic DNA, fresh young leaves of *S. brachista* were collected from Tianbao Mountain, Shangri-La Country, Yunnan Province, China. Additionally, we collected RNA from seven organs (roots, stems, young leaves, mature leaves, monoecious flowers, female flowers and male flowers) of Cushion willow plants. For each organ, three biological replicates were collected from different plants. The newly obtained materials were promptly frozen in liquid nitrogen. The related sequences were obtained from Kaitleai Mingjing Gene Technology (Beijing) Co., Ltd.

### Genome and transcriptome sequencing

For the genome, we extracted total DNA using the CTAB method for sequencing^16^. Before long-read sequencing, the DNA was purified by DNeasy Plant Mini Kit (Qiagen, Germantown, MD, USA). The purity and integrity of the DNA were subsequently assessed by 1% agarose gel electrophoresis. Qubit 2.0 fluorometer (Life Technologies, Carlsbad, CA, USA) was using to assess the concentration of DNA. Following the positive assessment result, we constructed a PacBio long-read library and generated 38.3 Gb (~2.11 million reads) of HiFi raw data on the PacBio Sequel II platform (Supplementary Table 1). We prepared a Hi-C library following standard protocols^17^. The library was subsequently sequenced on the Illumina NovaSeq 6000 platform. Approximately 49.7 Gb (330.98 million reads) of Hi-C raw data were obtained (Supplementary Table 1).

For the transcriptome sequencing of seven tissues, total RNA was extracted from 21 individual samples across seven organs using the Plant RNA Kit. The RNA quality and concentration were determined using a Nanodrop spectrophotometer and 1% agarose gel electrophoresis. In order to construct cDNA Illumina libraries, Oligo(dT) magnetic beads were utilized to eliminate rRNA, tRNA, and microRNA from the high-quality RNA. The mRNA was then reverse transcribed to yield cDNA by reverse transcriptase, and poly (A) and adapters were introduced. Finally, the cDNA was amplified by PCR to generate the cDNA library, which was subsequently sequenced on the Illumina NovaSeq. 6000 platform. Approximately 5 Gb of raw data were obtained from each sample (Supplementary Table 2). For Nanopore transcriptome sequencing, we first mixed the total cDNA from the five different organs (roots, mature leaves, young leaves, female flowers, and stems) of Cushion willow. The main step was adding a rapid adapter to each cDNA sequence to construct a 1D full-length library. This 1D library was run on an Oxford Nanopore PromethION. Finally, a total of ~16 Gb (16 million reads) of clean full-length transcripts was obtained (Supplementary Table 1).

### Haplotype genome assembly

**Fig. 1:**
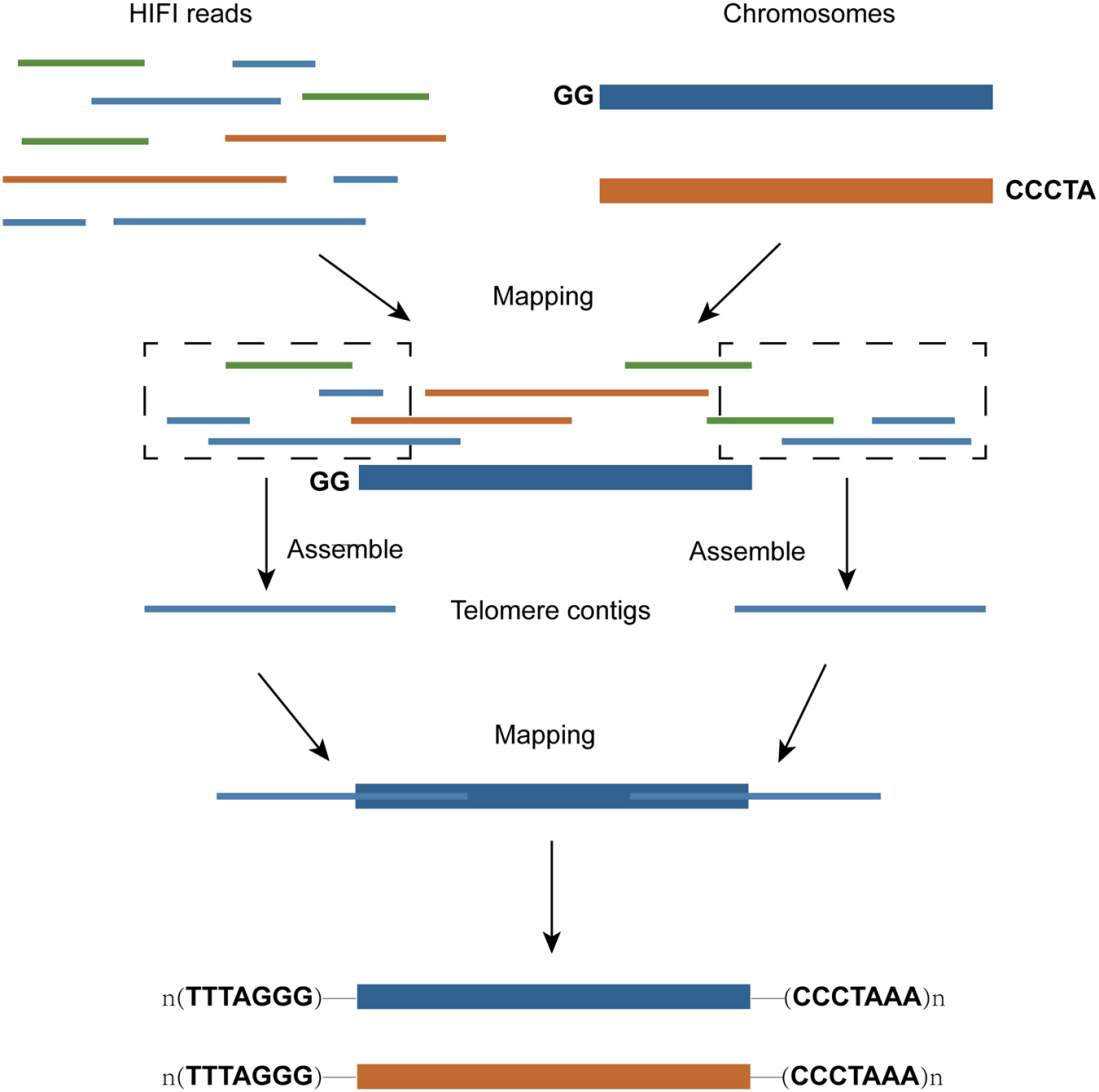
The schematic of assemble telomere to telomere.

We assembled the Cushion willow genome by combining PacBio single-molecule real-time long-read sequences, high-throughput chromatin conformation capture (Hi-C) sequences and Illumina short-read sequences from the National Center for Biotechnology Information (NCBI) Sequencing Read Archive (SRA) database (Project: PRJNA472210, Run: SRR9021434)^18^. First, we used HiFi data to initially assemble contigs using Hifiasm (v_0.16.1-r375)^19^. The Hi-C reads were compared with the contigs using Juicer^20^ and assisted in chromosome assembly using 3D-DNA (v_180922)^21^. We manually checked and adjusted the incorrect assemblies using Juicebox^22^. After two rounds of assisted assembly and manual correction, haplotype chromosome frames were generated. The gap in the chromosome was subsequently closed using TGS-GapCloser^23^ software based on the HiFi data (parameters:–ne–min_match 1000). Because of the chromosomes with unassembled telomeres or shorter telomeres, the HiFi reads were reused for comparison with the above-described assembled haplotype chromosomes by Minimap2^24^. Hifiasm software was used to reassemble the sequences near the telomeres, and the contigs obtained from the assembly were compared with the chromosomes to extend the chromosome telomeres outward and assemble a more complete telomere sequence TTTAGGG (Fig. 1)^25,26^. The assembled genome was subjected to two rounds of error correction using the software Nextpolish^27^ with Illumina data. Redundans^28^ was used to remove redundant sequences (rRNA fragments and low average coverage fragments)^25^. Finally, the two haplotype genomes were fully resolved at the chromosome level. The chromosome number and orientation were renamed according to the chromosome assembly of the Cushion willow published previously^15^. GetOrganelle (v_1.7.5)^29^ was used to assemble the chloroplast and mitochondrial genomes. The sizes of the chloroplasts and mitochondria were 155,612 bp and 630,081 bp, respectively (Supplementary Table 3).

### Gene identification and functional annotation

Before annotating the genomic information, we prepared two datasets for annotation. (1) We used publicly available homologous protein sequences from 17 species of the Salicaceae (*S. brachista*, *S. dunnii*, *S. purpurea*, *S. suchowensis*, *S. viminalis*, *Populus alba*, *P. alba* var. *pyramidalis*, *P. alba* x *P. glandulosa*, *P. davidiana* x *P. alba* var. *pyramidalis*, *P. deltoides*, *P. euphratica*, *P. ilicifolia*, *P. pruinosa*, *P. simonii*, *P. tremula*, *P. tremuloides*, *P. trichocarpa*) and *Arabidopsis thaliana* to combine 278,011 non-redundant protein sequences as homologous protein evidence for gene annotation. (2) The Oxford Nanopore Technology (ONT) transcriptome data were aligned to the reference genome using Minimap2^24^, followed by the use of Stringtie2 (v_2.2.1)^30^ to infer the transcript structure and assemble 85,880 transcripts. The Illumina RNA-seq data were downloaded from SRR7341541 in the SRA database^18^, which included 330,394 transcripts. Using PASA^31^, the above transcript data were merged into a set of transcript sequences containing 194,516 transcripts with structural annotations. Transposon elements were identified from scratch using EDTA^32^ (parameters:–sensitive 1–anno 1) to generate a TE library. Then, repeat regions in the genome were identified using RepeatMasker (http://www.repeatmasker.org/RepeatMasker/). A total of 1,138,238 repetitive sequences were identified, with a total length of 420,868,210 bp, accounting for 53.38% of the total genome length (Supplementary Table 4).

In the process of genome annotation, we undertook the following tasks: annotation, integration, and upgrading of genomes. Initially, we employed the MAKER2^33^ annotation pipeline to preliminarily integrate three types of annotations: *ab initio* gene prediction, homologous protein, and transcript evidence. We then masked repetitive sequence regions in the genome using RepeatMasker (default parameters). The AUGUSTUS^34^ software was utilized for *ab initio* prediction of the coding genes. The BLASTN and TBLASTX methods were employed for expression gene annotation, which was based on transcript evidence alignment with the genome. Exonerate^35^ was used to polish the annotation of expressed genes. Subsequently, AUGUSTUS was run again using the previous prediction results of expressed genes. Alternative splicing and UTR sequences were added to the predicted genes according to transcript evidence. The EVidenceModeler (EVM)^36^ was then run to integrate the results of gene annotation by MAKER2 and transcript evidence by PASA. Finally, the consistency gene annotation integrated results by EVM underwent two rounds of iterative upgrades using PASA. Upon obtaining these results, we removed coding frames containing stop codons, those without start codons or stop codons, and filtered overly short sequences less than 150 bp. Moreover, we used tRNAScan-SE^37^ to annotate tRNAs, Barrnap (v_0.9) (https://github.com/tseemann/barrnap) to annotate rRNAs and RfamScan^38^ to annotate various noncoding RNAs. In summary, we obtained 53,238 protein-coding genes, 1,414 rRNAs, 1,301 tRNAs and 1,216 ncRNAs (Supplementary Table 5).

After the genes were identified, their functional and structural characteristics needed to be annotated. This step involved the annotation of gene function using the homologous gene database eggNOG-mapper^39^ for GO and KEGG annotation. Furthermore, we obtained structural information about the genes through motif and domain annotations. This was achieved by employing InterProScan^40^ to compare structural domain similarities based on sub-databases of InterPro, such as the PRINTS, Pfam, SMART, and PANTHER databases. To determine the best alignment of the genes, Diamond^41^ was used to align protein sequences with several protein databases (Swiss_Prot, TrEMBL, NR and Arabidopsis) using the parameters identity > 30% and E value < 1e-5.

### Identification and expression analysis of allele genes

We identified alleles by the AlleleFinder pipeline (https://github.com/sc-zhang/AlleleFinder) based on the above high-quality haplotype genomes. Briefly, allele data were obtained from MCScanX^42^, GMAP^43^ and NCBI BLAST + based on two strategies: similarity and collinearity. First, using MCScanX, genes in syntenic regions were considered to be alleles. Then, the GMAP was used for further screening. Subsequently, alleles with more than 80% sequence similarity were recognized as pairs of alleles.

The RNA-seq data from the mature leaves of Cushion willow were used to analyze allele expression in both haplotypes. Initially, the adapters were trimmed, and the low-quality reads (<50 bp) were filtered out using Trimmomatic (v_0.39)^44^ software. Subsequently, the transcripts of the alleles were aligned to the homologous genome using HISAT2 (v_2.2.1)^45^ software. The fragments per kilobase of exon model per million mapped reads (FPKM) values were calculated to show allele expression levels via Stringtie2.

### Transcript alignment and assembly

To obtain better analysis results, it is essential to filter the RNA-seq raw data by removing the adapters and trimming the low-quality reads via Trimmomatic. Then, through the use of the quality control software FastQC (v_0.11.9) (http://www.bioinformatics.babraham.ac.uk/projects/fastqc/) and MultiQC (v_1.12)^46^, we obtained information on the clean data, including GC content and base sequence quality (Supplementary Table 2), of each sample. This information helped us to determine whether the results met the downstream analysis requirements.

The clean data from 21 samples were aligned, and the transcripts were assembled. First, HISAT2 was used to align the clean data with the reference genome (choosing the high-quality haploid genome A), thereby obtaining positional information about the transcripts. Then, using SAMtools (v_1.15)^47^, we obtained sorted binary bam files that revealed detailed information about the alignment results. Moreover, Stringtie2 was used to assemble the aligned reads to obtain the assembled transcripts of each sample. Overall, 28,587 non-redundant transcripts were assembled from seven organs.

**Fig. 2:**
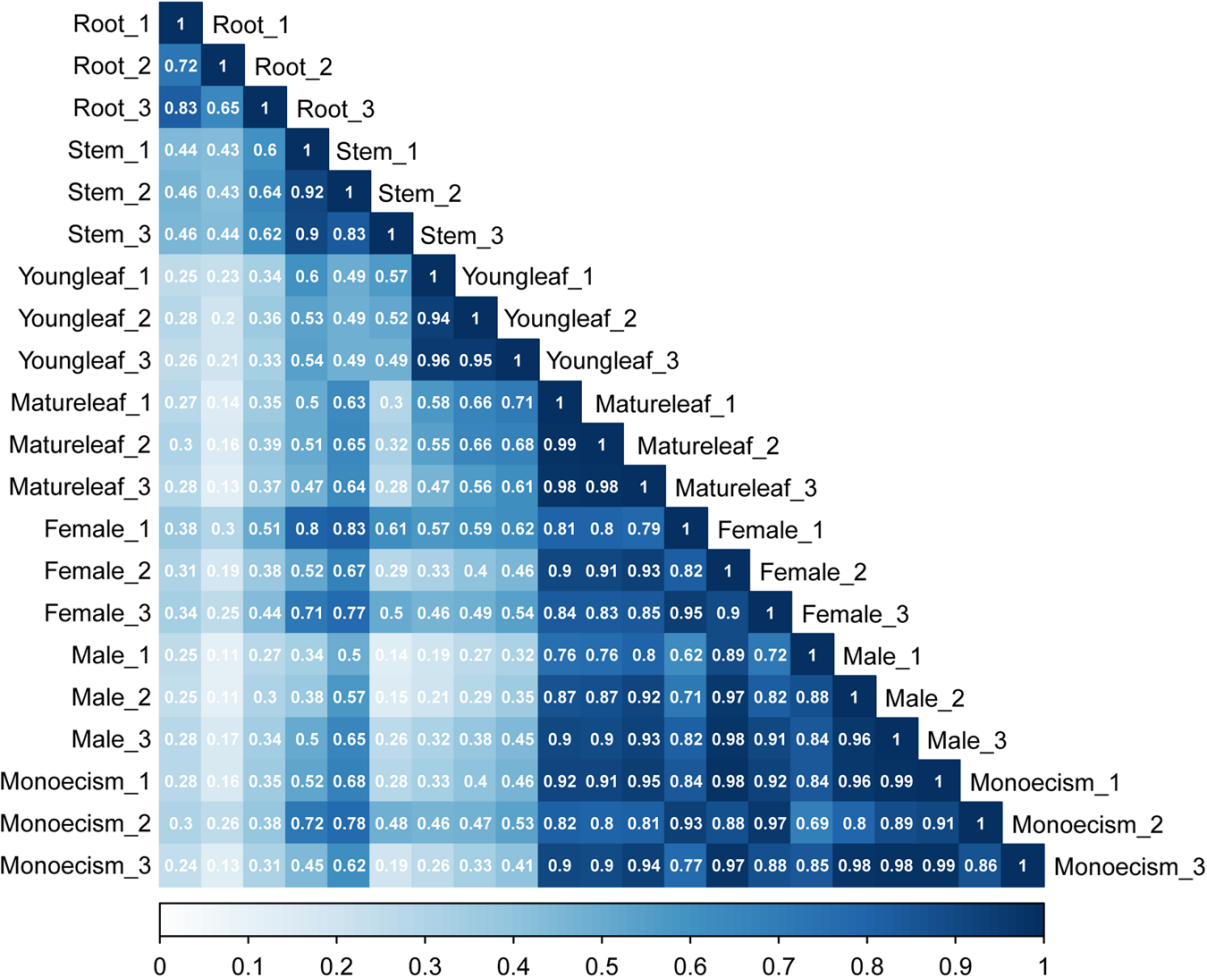
Correlation analysis of 21 RNA-seq samples from *S. brachista*. The dark blue color signifies greater similarity and repeatability between the two replicates.

It is necessary to conduct a repeated correlation test and eliminate samples with large differences in replicates to ensure the accuracy and value of the results. We computed the Pearson correlation coefficients among three biological replications based on the gene’s FPKM. The results revealed that the lowest Pearson’s correlation coefficient (r^2^) was 0.65, implying that all other correlation coefficients exceeded this value, this indicates robust replication among the biological replicates (Fig. 2).

### Determination of relative gene expression levels

The relative expression levels of each gene were calculated for various aspects, including high expression, organ-specific expression and differential expression. All calculations were based on the normalization of gene expression by FPKM values, which eliminated feature length and library size effects. We obtained gene expression tab files with FPKM values using the ‘-A’ parameter of Stringtie2. First, genes were screened and identified as expressed if FPKM > 0.3. Using a cutoff of FPKM > 20, distinctly high expression genes were detected in the root, stem, young leaf, mature leaf, monoecism flower, female flower and male flower of Cushion willow. Then, we subsequently compared and calculated the organ-specific expression of the genes via the R package Venn (v_1.10). Additionally, the differentially expressed genes (DEGs) were identified by the R package DESeq2 (v_1.32.0)^48^. Unlike FPKM, DESeq2 employs a negative binomial as its reference distribution and offers a unique normalization method called read count. Benjamini and Hochberg’s approach was used to adjust the resulting P values, thereby controlling the false discovery rate (FDR). The DEGs were designated as having an FDR < 0.01 and a log_2_ |fold change| >  = 1 according to DESeq2.

### GO and KEGG enrichment analysis

To predict the functions and pathways of genes across varying expression levels (highly expressed, organ-specifically expressed, differentially expressed), we annotated these genes using the GO and KEGG background files, which consist of three columns of information on gene ID, term and function/pathway extracted from the above genomic annotations. Using the R package clusterProfiler (v_4.0.5)^49^, the functions and pathways of genes at different expression levels were annotated by the GO and KEGG background files, respectively (pvalue: 0.05, padjustmethod: *Benjamini-Hochberg*). Finally, we compiled lists of the most notable gene annotations.

### Identification of AS events and APA sites

Based on the ONT full-length transcriptome data and the haplotype A genome of *S. brachista*, we performed an identification analysis of AS events and APA sites. First, we used the python script cdna_classifier.py in Pychopper2 (v_2.7.2) (https://github.com/epi2me-labs/pychopper) to trim and identify full-length transcripts. The full-length transcripts were self-corrected using the Flair (v_2.0.0)^50^ analysis process. Finally, the above full-length transcripts were utilized for the identification of variable AS events using the software package SUPPA2 (v_2.3)^51^ with default parameters. AS events were classified into seven categories: skipping exon (SE), mutually exclusive exon (MX), alternative 3’ splice site (A3), alternative 5’ splice site (A5), alternative first exon (AF), alternative last exon (AL) and retained intron (RI). APA sites were identified using LAPA (v_0.0.5)^52^.

## Data Records

The relevant raw data reported in this paper have been deposited in the National Genomics Data Center (NGDC)^53^, Beijing Institute of Genomics, Chinese Academy of Sciences/China National Center for Bioinformation under the BioProject accession number PRJCA022812 and PRJCA023075. The raw sequence data of genomic, including PacBio HiFi long-reads, Hi-C reads, have been deposited in the Genome Sequence Archive (GSA)^54^ at NGDC under the accession number CRA014642^55^. And raw sequence data of transcriptomic, which includes RNA-seq data from seven organs and Nanopore full-length transcript data, have also been deposited in the Genome Sequence Archive (GSA) at NGDC under the accession number CRA014607^56^. Genome assembly and annotation data has been deposited in the Genome Warehouse (GWH) in NGDC under the accession number GWHERDS00000000^57^. The allele genes data has been deposited in the figshare database^58^. The transcriptomic map data includes the FPKM of gene among seven tissues^59^, the GO and KEGG enrichment results of different expression levels^60^, AS events and APA sites^61^ can be store in the figshare database. The above data have been deposited in NCBI. The genomic raw data can be found in Sequence Read Archive (SRA) under the SRR32329603^62^ and SRR32329604^63^, while the assembly data has been deposited at GenBank under the accession JBLWMQ000000000^64^ and JBLWMR000000000^65^, and the genome’s annotation information is available in the figshare^66^. Additionally, the transcriptomic raw data have been deposited in the SRA under the accessions from SRR32340655 to SRR32340676^67–88^, and annotation files, read count files, FPKM files, and other processed files can be accessed through the GEO accession GSE289615^89^.

## Technical Validation

### Evaluation of the assembled and the annotated genome

The assembled haplotype genomes contained two high-quality haploid genomes, haplotype A and haplotype B. Haplotype A had 19 chromosomes with a genomic size of 401 Mb, while Haplotype B consisted of 19 chromosomes with a genomic size of 386 Mb. The GC content of both haploid genomes was 34.88% (Table 1). The scaffold N50 lengths of haplotype A and haplotype B were 22.63 Mb and 21.85 Mb, respectively (Table 1). Only one gap was found on the chromosome 15 of haplotype A and haplotype B (Supplementary Table 3).

The short reads and long reads were mapped to the assembled genome to evaluate genomic coverage by BWA^90^ and Minimap2. The RNA-seq reads were aligned to the assembled genome using HISAT2. After filtering out the non-primary alignment reads, we obtained a higher map ratio and coverage of sequencing reads (Table 2). We calculated the heterozygosity and single base error rate using Illumina reads, yielding a heterozygosity rate of approximately 0.0079% and an error rate of about 9.6e-06 (Q50). Using Hifi, Illumina and ONT data, we valued the GC content and sequencing depth under different GC content. The findings revealed no significant GC bias (Supplementary Fig. 1). The evaluation of the assembled genome was performed by BUSCO (v_2.0.1)^91^ with 1,440 groups from the lineage dataset embryophyta_odb10. The assembled genome BUSCO results indicated that complete core genes (including single-copy and multiple-copy genes) accounted for 96.0%, while the missing genes accounted for 3.2% (Table 3). This is consistent with previous WGD studies, suggesting that most of the genome of the Cushion willow has experienced duplication throughout its evolutionary history^15^. These BUSCO results showed a relatively high degree of gene completeness.

**Table 2:** The map ratio and coverage of reads obtained by different sequencing methods.

Dataset	Reads mapped (%)	Bases mapped (%)	> = 1× (%)	> = 5× (%)	> = 10× (%)	> = 20× (%)
Illumina	98.7	98.7	99.95	99.3	93.9	43.5
HiFi	99.6	99.6	99.95	99.9	99.7	98.3
ONT	66.7	95.3	99.9	99.3	96.7	84.1
RNA-seq	92.7	97.8	22.3	13.1	10.7	8.4

**Table 3:** BUSCO evaluation of the assembled genome and the annotated proteome.

Type	Genome	Proteome
Complete BUSCOs (C)	96.0%	97.7%
Complete and single-copy BUSCOs (S)	6.5%	2.4%
Complete and duplicated BUSCOs (D)	89.5%	95.3%
Fragmented BUSCOs (F)	0.8%	0.9%
Missing BUSCOs (M)	3.2%	1.9%
Total BUSCO groups	1440	1440

**Fig. 3:**
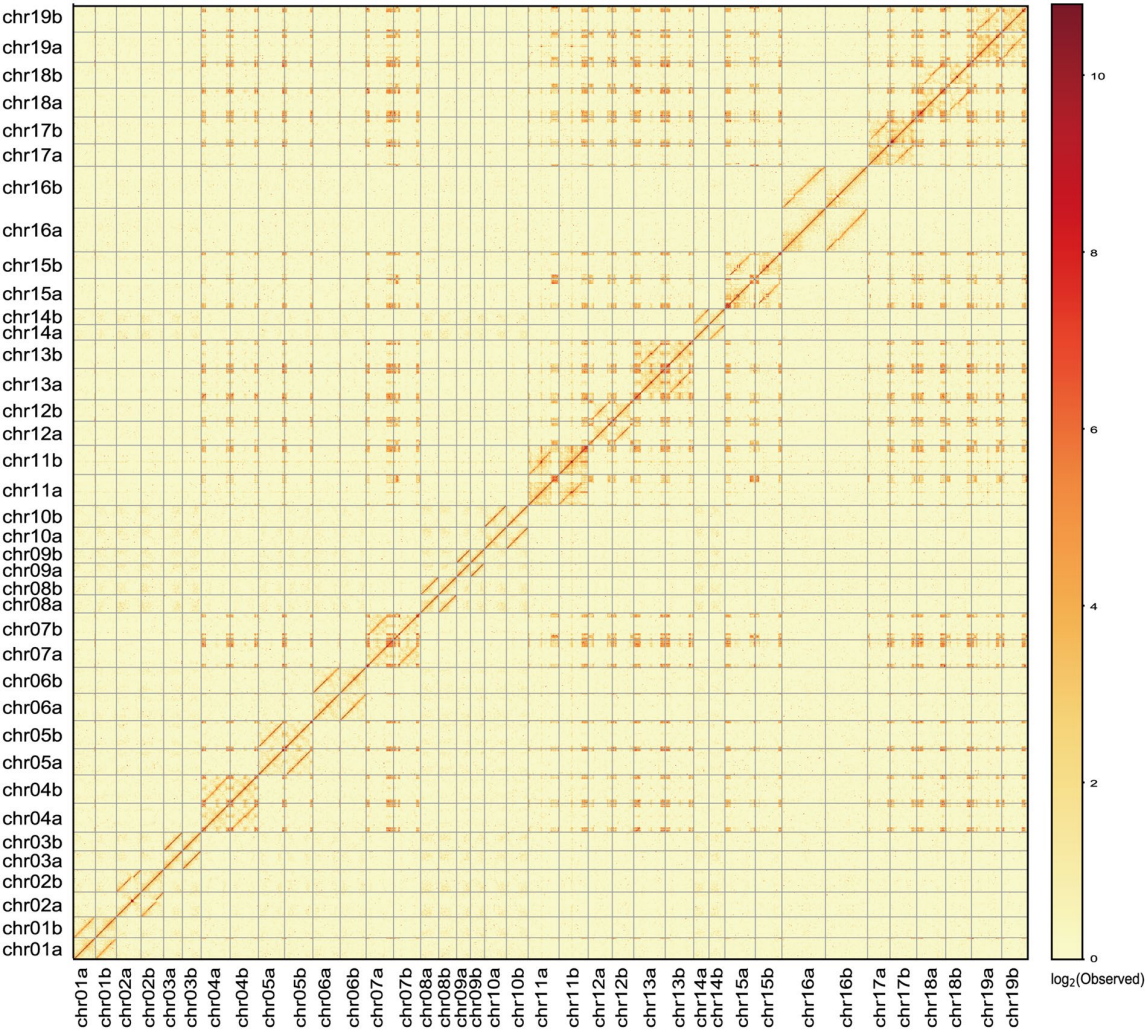
Hi-C heatmap of the two final assembly haploids. The colors from yellow (low) to red (high) indicate the strength of the interaction.

By mapping the Hi-C data to the final assembled genome with Juicer, we observed strong chromosomal clustering and assembly (Fig. 3). A comparison of sequences from the published genome and the two haploids was conducted using Minimap2. The results indicated that the chromosome order was identical in both instances (Supplementary Fig. 2). Characteristic sequences such as telomeres, tandem repeats, and 5S and 8-5.8-28S rDNAs were identified on the chromosomes (Supplementary Fig. 3). In summary, the two haplotypes exhibited comprehensive and well-assembled assemblies. The high-quality Cushion willow genome can serve as a reference for studies in the future.

A total of 52,715 protein-coding genes were identified across various databases, accounting for 99.02% of the total (Table 4). The assessment of the annotated proteome was conducted using BUSCO, revealing that 97.7% of the complete BUSCOs were present in the annotated proteome (Table 3).

**Table 4:** Summary of predicted gene annotations in the *S*. *brachista* genome.

Annotation method	Database	Gene number	Percentage (%)
	All protein-coding genes	53,238	100
Annotated by eggNOG-mapper	GO	26,390	49.57
	KEEG pathway	15,063	28.29
	KEEG_KO	24,413	45.86
	eggNOG	47,656	89.52
	COG	51,326	96.41
	EC	10,635	19.98
	Unannotated	1,912	3.59
Annotated by Diamond	Swiss_Prot	39,503	74.20
	TrEMBL	51,799	97.30
	NR	51,762	97.23
	*A. thaliana*	47,543	89.30
	Unannotated	1,362	2.56
Annotated by InterProScan	Pfam	43,494	81.70
	CDD	19,116	35.91
	PRINTS	7,680	14.43
	PIRSF	3,575	6.71
	PANTHER	50,189	94.27
	Interpro	45,709	85.85
	Phobius	19,088	35.85
	Gene3D	36,202	68.00
	SUPERFAMILY	34,165	64.17
	TIGRFAM	5,643	10.60
	MobiDBLite	23,688	44.49
	Coils	8,601	16.16
	TMHMM	13,003	24.42
	SMART	17,532	32.93
	Unannotated	950	1.78
Total	Annotated	52,715	99.02
	Unannotated	523	0.98

To characterize the assembly of alleles in the two haplotype genomes, we performed allele genes by the AlleleFinder pipeline. In total, 23,744 allele genes were identified, comprising 1,459 paralog and 647 tandem genes (Supplementary Table 6). The 17,885 pair of allele genes expressed.

### Evaluation of the assembled transcriptome

**Fig. 4:**
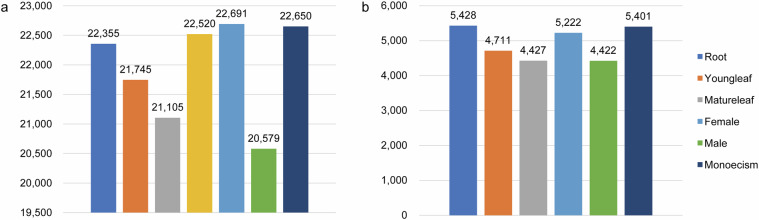
The expression levels of genes across seven organs of *S. brachista*. (**a**) The gene profiles of seven various organs (FPKM > 0.3). (**b**) Genes with high expression levels in seven different organs. (FPKM > 20).

**Fig. 5:**
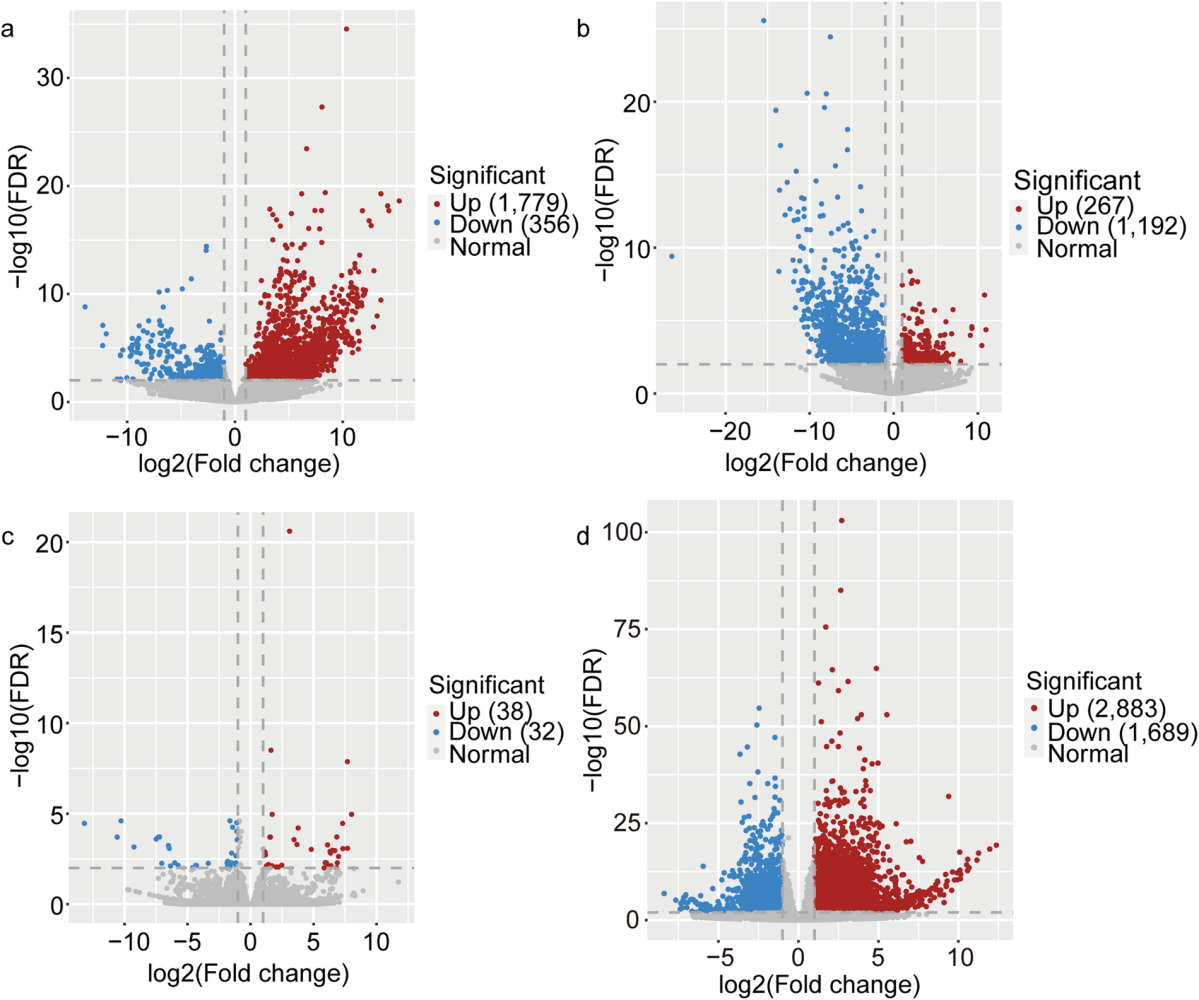
Volcano plots of DEGs in each pairwise comparison of different flowering lines and young leaves vs. mature leaves. (**a**) Female vs. Male. (**b**) Female vs. Monoecism. (**c**) Male vs. Monoecism. (**d**) Young leaf vs. Mature leaf. Blue is up, red is down, and gray is not significant.

**Fig. 6:**
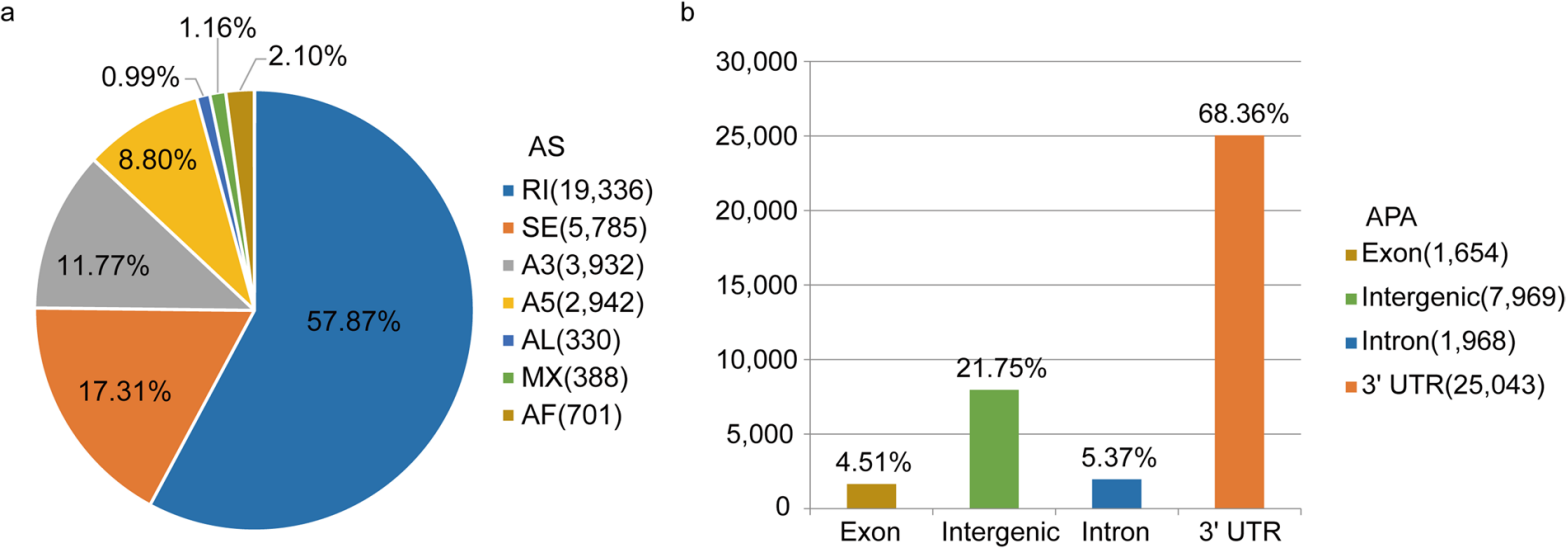
The types and numbers of AS events and APA sites. (**a**) Alternative splicing events. SE, skipping exon; MX, mutually exclusive exon; A3, alternative 3’ splice site; A5, alternative 5’ splice site; AF, alternative first exon; AL, alternative last exon; RI, retained intron. (**b**) APA, Alternative polyadenylation sites.

The clean data from 21 samples were aligned to haplotype genome A using HISAT2, yielding an average mapping rate of 86.63%. This suggests a significant proportion of the mapped clean reads (Supplementary Table 7). Following the use of the reference assembly, we obtained 28,587 non-redundant transcripts. The mean number of expressed genes per tissue was determined as 21,949 using the threshold FPKM > 0.3, with highly expressed genes comprising 22.62% of all expressed genes (FPKM > 20) (Fig. 4). Using Venn showed the organ-specific expression genes (Supplementary Fig. 4). Furthermore, we identified 17,387 DEGs across seven organs (Supplementary Fig. 5). We classified the expressed genes based on GO terms and KEGG pathways. Our analysis showed that genes with different expression levels were enriched in various metabolic pathways and functions, with a significant proportion of DEGs being associated with specific pathways and functions in both KEGG and GO (Fig. 5). Full-length transcript data can identify more accurately AS and APA sites, which are crucial for researching the regulatory mechanisms of gene expression. Using the ONT full-length transcripts, we discovered a total of 33,414 AS events, including RI, 19,336; SE, 5,785; A3, 3,932; A5, 2,942; AL, 330; MX, 355; and AF, 701 (Fig. 6a). Through the use of LAPA, we identified 36,634 poly (A) sites distributed across intergenic regions, 3’ UTRs, introns, and exons (Fig. 6b).

The aforementioned evidence collectively indicates that the transcriptomic map is both dependable and precise. The preliminary transcriptomic map of the Cushion willow offers invaluable resources for elucidating the adaptation strategies of this species to environments at extremely high altitudes.

## Supplementary information

Supplementary information of A haplotype-resolved genome assembly and gene expression map of Cushion willow

## Data Availability

No custom script was used in this work. Data processing was performed using the relevant bioinformatics software protocols and manuals. The version and parameters of the software used are described in the Methods section.

## References

[1] Argus, G. W., Eckenwalder, J. E., Kiger, R. W. Salicaceae. (2010).

[2] Skvortsov, A. K. Willows of Russia and adjacent countries. *University of Joensuu, Joensuu, Finland* (1999).

[3] Fang, Z. F., Zhao, S. D. & Skvortsov, A. K. *Flora of China: Salicaceae*. 139–274 (Science Press, 1999).

[4] Karp, A. & Shield, I. Bioenergy from plants and the sustainable yield challenge. *New Phytol* **179**, 15–32, 10.1111/j.1469-8137.2008.02432.x (2008). 18422906 10.1111/j.1469-8137.2008.02432.x

[5] Karp, A. *et al*. Genetic improvement of willow for bioenergy and biofuels. *J Integr Plant Biol* **53**, 151–165, 10.1111/j.1744-7909.2010.01015.x (2011). 21205181 10.1111/j.1744-7909.2010.01015.x

[6] Pucholt, P., Ronnberg-Wastljung, A. C. & Berlin, S. Single locus sex determination and female heterogamety in the basket willow (*Salix viminalis* L.). *Heredity (Edinb)* **114**, 575–583, 10.1038/hdy.2014.125 (2015). 25649501 10.1038/hdy.2014.125 PMC4434249

[7] Zhou, R. *et al*. Characterization of a large sex determination region in *Salix purpurea* L. (Salicaceae). *Mol Genet Genomics* **293**, 1437–1452, 10.1007/s00438-018-1473-y (2018). 30022352 10.1007/s00438-018-1473-y

[8] Sanderson, B. J. *et al*. Sex determination through X-Y heterogamety in *Salix nigra*. *Heredity (Edinb)* **126**, 630–639, 10.1038/s41437-020-00397-3 (2021). 33510464 10.1038/s41437-020-00397-3 PMC8115673

[9] Wilkerson, D. G., Taskiran, B., Carlson, C. H. & Smart, L. B. Mapping the sex determination region in the *Salix* F1 hybrid common parent population confirms a ZW system in six diverse species. *G3 (Bethesda)* **12**10.1093/g3journal/jkac071 (2022). 10.1093/g3journal/jkac071 PMC9157088 35333299

[10] Hu, N. *et al*. Evolution of a ZW sex chromosome system in willows. *Nat Commun* **14**, 7144, 10.1038/s41467-023-42880-5 (2023). 37932261 10.1038/s41467-023-42880-5 PMC10628195

[11] Qiao, Q. *et al*. Transcriptome sequencing of *Crucihimalaya himalaica* (Brassicaceae) reveals how *Arabidopsis* close relative adapt to the Qinghai-Tibet Plateau. *Sci Rep* **6**, 21729, 10.1038/srep21729 (2016). 26906946 10.1038/srep21729 PMC4764839

[12] Lv, M. *et al*. Effect of UV-B radiation on growth, flavonoid and podophyllotoxin accumulation, and related gene expression in *Sinopodophyllum hexandrum*. *Plant Biol (Stuttg)* **23**(Suppl 1), 202–209, 10.1111/plb.13226 (2021). 33280221 10.1111/plb.13226

[13] Li, M. F. *et al*. Mapping podophyllotoxin biosynthesis and growth-related transcripts with high elevation in *Sinopodophyllum hexandrum*. *Ind. Crops Prod* **124**, 510–518, 10.1016/j.indcrop.2018.08.007 (2018).

[14] Guo, X. *et al*. The genomes of two *Eutrema* species provide insight into plant adaptation to high altitudes. *DNA Res* **25**, 307–315, 10.1093/dnares/dsy003 (2018). 29394339 10.1093/dnares/dsy003 PMC6014361

[15] Chen, J. H. *et al*. Genome-wide analysis of Cushion willow provides insights into alpine plant divergence in a biodiversity hotspot. *Nat Commun* **10**, 5230, 10.1038/s41467-019-13128-y (2019). 31745089 10.1038/s41467-019-13128-y PMC6864086

[16] Doyle, J. D. J. L. A rapid DNA isolation procedure for small quantities of fresh leaf tissue. *Phytochem Bull* **19**, 11–15 (1987).

[17] Belton, J. M. *et al*. Hi-C: a comprehensive technique to capture the conformation of genomes. *Methods* **58**, 268–276, 10.1016/j.ymeth.2012.05.001 (2012). 22652625 10.1016/j.ymeth.2012.05.001 PMC3874846

[18] *NCBI Sequence Read Archive* https://www.ncbi.nlm.nih.gov/sra/SRR9021434 (2019).

[19] Cheng, H., Concepcion, G. T., Feng, X., Zhang, H. & Li, H. Haplotype-resolved *de novo* assembly using phased assembly graphs with hifiasm. *Nat Methods* **18**, 170–175, 10.1038/s41592-020-01056-5 (2021). 33526886 10.1038/s41592-020-01056-5 PMC7961889

[20] Durand, N. C. *et al*. Juicer provides a one-click system for analyzing loop-resolution Hi-C experiments. *Cell Syst* **3**, 95–98, 10.1016/j.cels.2016.07.002 (2016). 27467249 10.1016/j.cels.2016.07.002 PMC5846465

[21] Dudchenko, O. *et al*. *De novo* assembly of the *Aedes aegypti* genome using Hi-C yields chromosome-length scaffolds. *Science* **356**, 92–95, 10.1126/science.aal3327 (2017). 28336562 10.1126/science.aal3327 PMC5635820

[22] Durand, N. C. *et al*. Juicebox provides a visualization system for Hi-C contact maps with unlimited zoom. *Cell Syst* **3**, 99–101, 10.1016/j.cels.2015.07.012 (2016). 27467250 10.1016/j.cels.2015.07.012 PMC5596920

[23] Xu, M. *et al*. TGS-GapCloser: A fast and accurate gap closer for large genomes with low coverage of error-prone long reads. *Gigascience* **9**10.1093/gigascience/giaa094 (2020). 10.1093/gigascience/giaa094 PMC7476103 32893860

[24] Li, H. Minimap2: pairwise alignment for nucleotide sequences. *Bioinformatics* **34**, 3094–3100, 10.1093/bioinformatics/bty191 (2018). 29750242 10.1093/bioinformatics/bty191 PMC6137996

[25] Chang, Y., Zhang, R., Ma, Y. & Sun, W. A haplotype-resolved genome assembly of *Rhododendron vialii* based on PacBio HiFi reads and Hi-C data. *Sci Data* **10**, 451, 10.1038/s41597-023-02362-1 (2023). 37438373 10.1038/s41597-023-02362-1 PMC10338486

[26] He, L. *et al*. Allopolyploidization from two dioecious ancestors leads to recurrent evolution of sex chromosomes. *Nat Commun* **15**, 6893, 10.1038/s41467-024-51158-3 (2024). 39134553 10.1038/s41467-024-51158-3 PMC11319354

[27] Hu, J., Fan, J., Sun, Z. & Liu, S. NextPolish: a fast and efficient genome polishing tool for long-read assembly. *Bioinformatics* **36**, 2253–2255, 10.1093/bioinformatics/btz891 (2020). 31778144 10.1093/bioinformatics/btz891

[28] Pryszcz, L. P. & Gabaldon, T. Redundans: an assembly pipeline for highly heterozygous genomes. *Nucleic Acids Res* **44**, e113, 10.1093/nar/gkw294 (2016). 27131372 10.1093/nar/gkw294 PMC4937319

[29] Jin, J. J. *et al*. GetOrganelle: a fast and versatile toolkit for accurate *de novo* assembly of organelle genomes. *Genome Biol* **21**, 241, 10.1186/s13059-020-02154-5 (2020). 32912315 10.1186/s13059-020-02154-5 PMC7488116

[30] Pertea, M. *et al*. StringTie enables improved reconstruction of a transcriptome from RNA-seq reads. *Nat Biotechnol* **33**, 290–295, 10.1038/nbt.3122 (2015). 25690850 10.1038/nbt.3122 PMC4643835

[31] Haas, B. J. *et al*. Improving the *Arabidopsis* genome annotation using maximal transcript alignment assemblies. *Nucleic Acids Res* **31**, 5654–5666, 10.1093/nar/gkg770 (2003). 14500829 10.1093/nar/gkg770 PMC206470

[32] Ou, S. *et al*. Benchmarking transposable element annotation methods for creation of a streamlined, comprehensive pipeline. *Genome Biol* **20**, 275, 10.1186/s13059-019-1905-y (2019). 31843001 10.1186/s13059-019-1905-y PMC6913007

[33] Cantarel, B. L. *et al*. MAKER: An easy-to-use annotation pipeline designed for emerging model organism genomes. *Genome Res* **18**, 188–196, 10.1101/gr.6743907 (2008). 18025269 10.1101/gr.6743907 PMC2134774

[34] Stanke, M., Diekhans, M., Baertsch, R. & Haussler, D. Using native and syntenically mapped cDNA alignments to improve *de novo* gene finding. *Bioinformatics* **24**, 637–644, 10.1093/bioinformatics/btn013 (2008). 18218656 10.1093/bioinformatics/btn013

[35] Slater, G. S. & Birney, E. Automated generation of heuristics for biological sequence comparison. *BMC Bioinformatics* **6**, 31, 10.1186/1471-2105-6-31 (2005). 15713233 10.1186/1471-2105-6-31 PMC553969

[36] Haas, B. J. *et al*. Automated eukaryotic gene structure annotation using EVidenceModeler and the Program to Assemble Spliced Alignments. *Genome Biol* **9**, R7, 10.1186/gb-2008-9-1-r7 (2008). 18190707 10.1186/gb-2008-9-1-r7 PMC2395244

[37] Lowe, T. M. & Eddy, S. R. tRNAscan-SE: a program for improved detection of transfer RNA genes in genomic sequence. *Nucleic Acids Res* **25**, 955–964, 10.1093/nar/25.5.955 (1997). 9023104 10.1093/nar/25.5.955 PMC146525

[38] Nawrocki, E. P. *et al*. Rfam 12.0: updates to the RNA families database. *Nucleic Acids Res* **43**, D130–137, 10.1093/nar/gku1063 (2015). 25392425 10.1093/nar/gku1063 PMC4383904

[39] Huerta-Cepas, J. *et al*. Fast genome-wide functional annotation through orthology assignment by eggNOG-Mapper. *Mol Biol Evol* **34**, 2115–2122, 10.1093/molbev/msx148 (2017). 28460117 10.1093/molbev/msx148 PMC5850834

[40] Jones, P. *et al*. InterProScan 5: genome-scale protein function classification. *Bioinformatics* **30**, 1236–1240, 10.1093/bioinformatics/btu031 (2014). 24451626 10.1093/bioinformatics/btu031 PMC3998142

[41] Buchfink, B., Xie, C. & Huson, D. H. Fast and sensitive protein alignment using DIAMOND. *Nat Methods* **12**, 59–60, 10.1038/nmeth.3176 (2015). 25402007 10.1038/nmeth.3176

[42] Wang, Y. *et al*. *MCScanX*: a toolkit for detection and evolutionary analysis of gene synteny and collinearity. *Nucleic Acids Res* **40**, e49, 10.1093/nar/gkr1293 (2012). 22217600 10.1093/nar/gkr1293 PMC3326336

[43] Wu, T. D. & Watanabe, C. K. GMAP: a genomic mapping and alignment program for mRNA and EST sequences. *Bioinformatics* **21**, 1859–1875, 10.1093/bioinformatics/bti310 (2005). 15728110 10.1093/bioinformatics/bti310

[44] Bolger, A. M., Lohse, M. & Usadel, B. Trimmomatic: a flexible trimmer for Illumina sequence data. *Bioinformatics* **30**, 2114–2120, 10.1093/bioinformatics/btu170 (2014). 24695404 10.1093/bioinformatics/btu170 PMC4103590

[45] Kim, D., Langmead, B. & Salzberg, S. L. HISAT: a fast spliced aligner with low memory requirements. *Nat Methods* **12**, 357–360, 10.1038/nmeth.3317 (2015). 25751142 10.1038/nmeth.3317 PMC4655817

[46] Ewels, P., Magnusson, M., Lundin, S. & Kaller, M. MultiQC: summarize analysis results for multiple tools and samples in a single report. *Bioinformatics* **32**, 3047–3048, 10.1093/bioinformatics/btw354 (2016). 27312411 10.1093/bioinformatics/btw354 PMC5039924

[47] Danecek, P. *et al*. Twelve years of SAMtools and BCFtools. *GigaScience* **10**10.1093/gigascience/giab008 (2021). 10.1093/gigascience/giab008 PMC7931819 33590861

[48] Love, M. I., Huber, W. & Anders, S. Moderated estimation of fold change and dispersion for RNA-seq data with DESeq2. *Genome Biol* **15**, 550, 10.1186/s13059-014-0550-8 (2014). 25516281 10.1186/s13059-014-0550-8 PMC4302049

[49] Wu, T. *et al*. clusterProfiler 4.0: A universal enrichment tool for interpreting omics data. *Innovation (Camb)* **2**, 100141, 10.1016/j.xinn.2021.100141 (2021). 34557778 10.1016/j.xinn.2021.100141 PMC8454663

[50] Tang, A. D. *et al*. Full-length transcript characterization of *SF3B1* mutation in chronic lymphocytic leukemia reveals downregulation of retained introns. *Nat Commun* **11**, 1438, 10.1038/s41467-020-15171-6 (2020). 32188845 10.1038/s41467-020-15171-6 PMC7080807

[51] Trincado, J. L. *et al*. SUPPA2: fast, accurate, and uncertainty-aware differential splicing analysis across multiple conditions. *Genome Biol* **19**, 40, 10.1186/s13059-018-1417-1 (2018). 29571299 10.1186/s13059-018-1417-1 PMC5866513

[52] Çelik, M. H. & Mortazavi, A. Analysis of alternative polyadenylation from long-read or short-read RNA-seq with LAPA. *bioRxiv*10.1101/2022.11.08.515683 (2022).

[53] Members, C.-N. & Partners. Database resources of the National Genomics Data Center, China national center for bioinformation in 2024. *Nucleic Acids Res* **52**, D18–D32, 10.1093/nar/gkad1078 (2024). 38018256 10.1093/nar/gkad1078 PMC10767964

[54] Chen, T. *et al*. The Genome Sequence Archive family: toward explosive data growth and diverse data types. *Genomics Proteomics Bioinformatics* **19**, 578–583, 10.1016/j.gpb.2021.08.001 (2021). 34400360 10.1016/j.gpb.2021.08.001 PMC9039563

[55] *NGDC Genome Sequence Archive* https://ngdc.cncb.ac.cn/gsa/browse/CRA014642 (2024).

[56] *NGDC Genome Sequence Archive* https://ngdc.cncb.ac.cn/gsa/browse/CRA014607 (2024).

[57] *NGDC Genome Warehouse* https://ngdc.cncb.ac.cn/gwh/Assembly/GWHERDS00000000 (2024).

[58] Wang, J. A dataset of allele genes of *Salix brachista*, *figshare*, 10.6084/m9.figshare.27013480.v1 2024).

[59] Wang, J. Expression genes (FPKM>0.3) of *Salix brachista*. *figshare*10.6084/m9.figshare.27013468.v1 (2024).

[60] Wang, J. The KEGG and GO results of different expression levels in *Salix brachista*. *figshare*10.6084/m9.figshare.27013810.v1 (2024).

[61] Wang, J. Alternative splicing (AS) and alternative polyadenylation (APA) sites of *Salix brachista*. *figshare*10.6084/m9.figshare.27013288.v1 (2024).

[62] *NCBI Sequence Read Arichive* http://identifiers.org/ncbi/insdc.sra:SRR32329603 (2025).

[63] *NCBI Sequence Read Arichive* http://identifiers.org/ncbi/insdc.sra:SRR32329604 (2025).

[64] *NCBI GenBank* https://identifiers.org/ncbi/insdc:JBLWMQ010000000 (2025).

[65] *NCBI GenBank* https://identifiers.org/ncbi/insdc:JBLWMR010000000 (2025).

[66] Wang, J. The annotation files of *Salix brachista* genome, *figshare*, 10.6084/m9.figshare.28822880.v1 (2025).

[67] *NCBI Sequence Read Archive* http://identifiers.org/ncbi/insdc.sra:SRR32340655 (2025).

[68] *NCBI Sequence Read Arichive* http://identifiers.org/ncbi/insdc.sra:SRR32340656 (2025).

[69] *NCBI Sequence Read Archive* http://identifiers.org/ncbi/insdc.sra:SRR32340657 (2025).

[70] *NCBI Sequence Read Arichive* http://identifiers.org/ncbi/insdc.sra:SRR32340658 (2025).

[71] *NCBI Sequence Read Arichive* http://identifiers.org/ncbi/insdc.sra:SRR32340659 (2025).

[72] *NCBI Sequence Read Archive* http://identifiers.org/ncbi/insdc.sra:SRR32340660 (2025).

[73] *NCBI Sequence Read Archive* http://identifiers.org/ncbi/insdc.sra:SRR32340661 (2025).

[74] *NCBI Sequence Read Archive* http://identifiers.org/ncbi/insdc.sra:SRR32340662 (2025).

[75] *NCBI Sequence Read Archive* http://identifiers.org/ncbi/insdc.sra:SRR32340663 (2025).

[76] *NCBI Sequence Read Archive* http://identifiers.org/ncbi/insdc.sra:SRR32340664 (2025).

[77] *NCBI Sequence Read Archive* http://identifiers.org/ncbi/insdc.sra:SRR32340665 (2025).

[78] *NCBI Sequence Read Archive* http://identifiers.org/ncbi/insdc.sra:SRR32340666 (2025).

[79] *NCBI Sequence Read Archive* http://identifiers.org/ncbi/insdc.sra:SRR32340667 (2025).

[80] *NCBI Sequence Read Archive* http://identifiers.org/ncbi/insdc.sra:SRR32340668 (2025).

[81] *NCBI Sequence Read Archive* http://identifiers.org/ncbi/insdc.sra:SRR32340669 (2025).

[82] *NCBI Sequence Read Archive* http://identifiers.org/ncbi/insdc.sra:SRR32340670 (2025).

[83] *NCBI Sequence Read Archive* http://identifiers.org/ncbi/insdc.sra:SRR32340671 (2025).

[84] *NCBI Sequence Read Archive* http://identifiers.org/ncbi/insdc.sra:SRR32340672 (2025).

[85] *NCBI Sequence Read Archive* http://identifiers.org/ncbi/insdc.sra:SRR32340673 (2025).

[86] *NCBI Sequence Read Archive* http://identifiers.org/ncbi/insdc.sra:SRR32340674 (2025).

[87] *NCBI Sequence Read Archive* http://identifiers.org/ncbi/insdc.sra:SRR32340675 (2025).

[88] *NCBI Sequence Read Archive* http://identifiers.org/ncbi/insdc.sra:SRR32340676 (2025).

[89] *NCBI GEO* http://identifiers.org/geo/GSE289615 (2025).

[90] Li, H. Aligning sequence reads, clone sequences and assembly contigs with BWA-MEM. *arXiv: Genomics* (2013).

[91] Simao, F. A., Waterhouse, R. M., Ioannidis, P., Kriventseva, E. V. & Zdobnov, E. M. BUSCO: assessing genome assembly and annotation completeness with single-copy orthologs. *Bioinformatics* **31**, 3210–3212, 10.1093/bioinformatics/btv351 (2015). 26059717 10.1093/bioinformatics/btv351

