# Supplementary material for: A haplotype-resolved genome assembly and gene expression map of Cushion willow
Source: Sci Data. 2025 May 13;12:785. doi: 10.1038/s41597-025-05132-3 (PMC12075806; doi:10.1038/s41597-025-05132-3)
Supplement: Supplementary file 1 — Supplementary information of A haplotype-resolved genome assembly and gene expression map of Cushion willow [file 41597_2025_5132_MOESM1_ESM.pdf]

A haplotype-resolved genome assembly and gene expression map of Cushion willow

Supplementary Fig. 1. The scatterplot of GC and coverage depth distribution of HiFi data (a), the Illumina data (b) and ONT data (c).

Supplementary Fig. 2. Dot-plot of synteny blocks between the published genome and two haplotypes within *S. brachista*.

Supplementary Fig. 3. Bar plots of distribution of several characteristic sequences across 38 chromosomes.

Supplementary Fig. 4. A Venn of specific-organ expression genes.

Supplementary Fig. 5. A heatmap of 17,387 DEGs among seven tissues.

Supplementary Table 1. Statistics of the long-read HiFi data, Hi-C data and full-length transcripts data.

Supplementary Table 2. Transcriptome data of each sample statistics.

Supplementary Table 3. Statistics of the chromosomal level assembly *S. brachista*.

Supplementary Table 4. Repeat annotations of the *S. brachista* genome assembly.

Supplementary Table 5. Annotations of the *S. brachista* genome assembly.

Supplementary Table 6. The identification results of the *S. brachista* allele gene**s.**

Supplementary Table 7. The alignment rate of clean data in 21 samples.

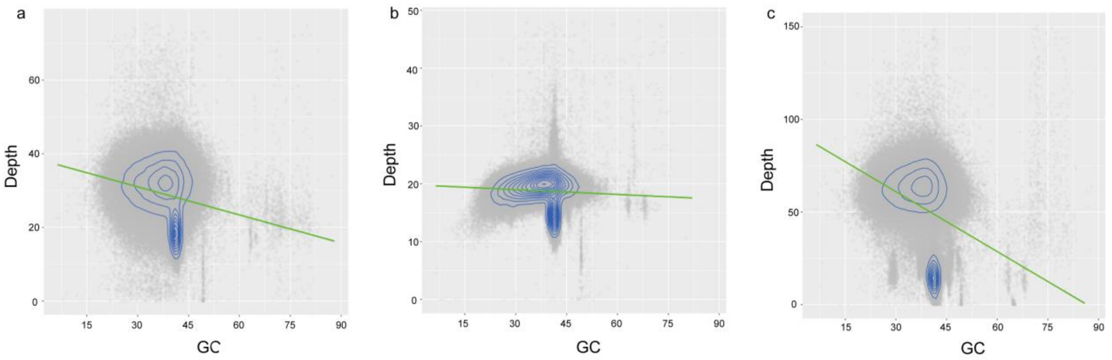

Supplementary Fig. 1. The scatterplot of GC and coverage depth distribution of HiFi data (a), the Illumina data (b) and ONT data (c).

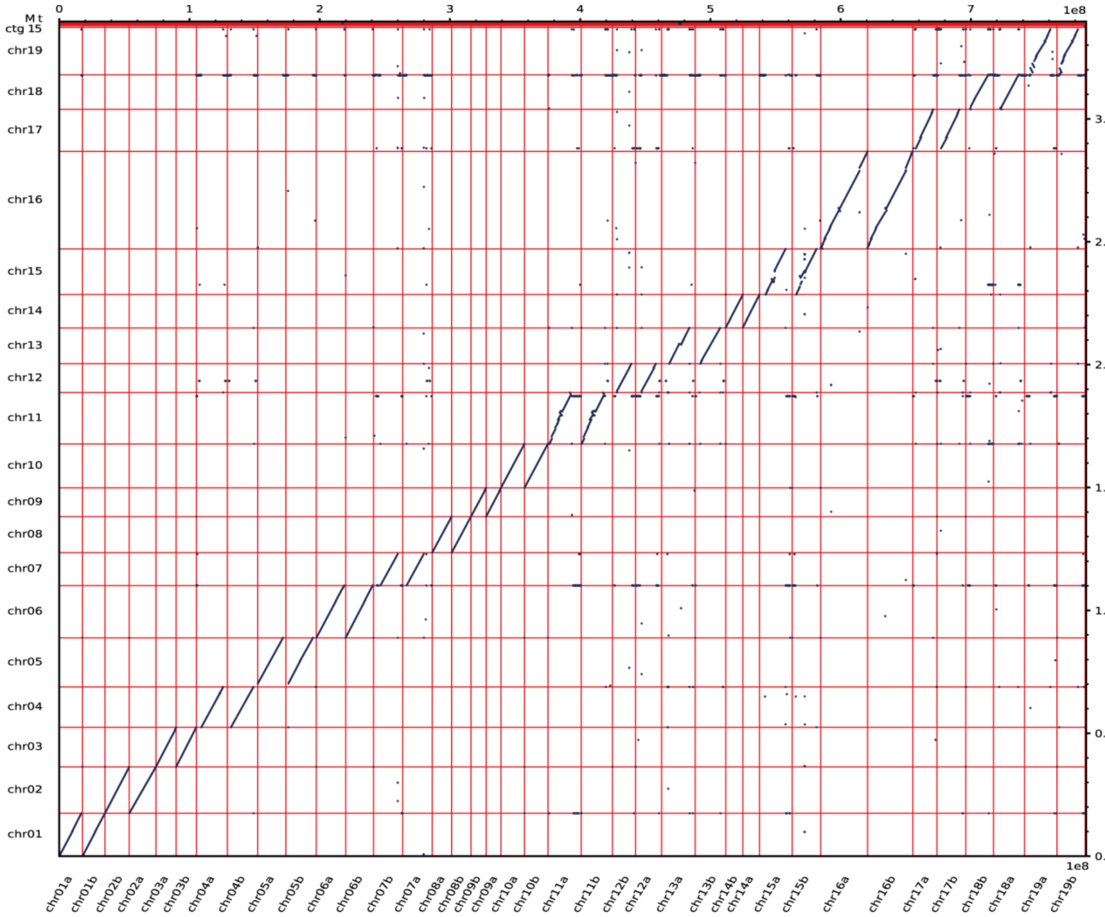

Supplementary Fig. 2. Dot-plot of synteny blocks between the published genome and two haplotypes within *S. brachista*. The x-axis represents the two haploid genomes and the y-axis is the published genome.

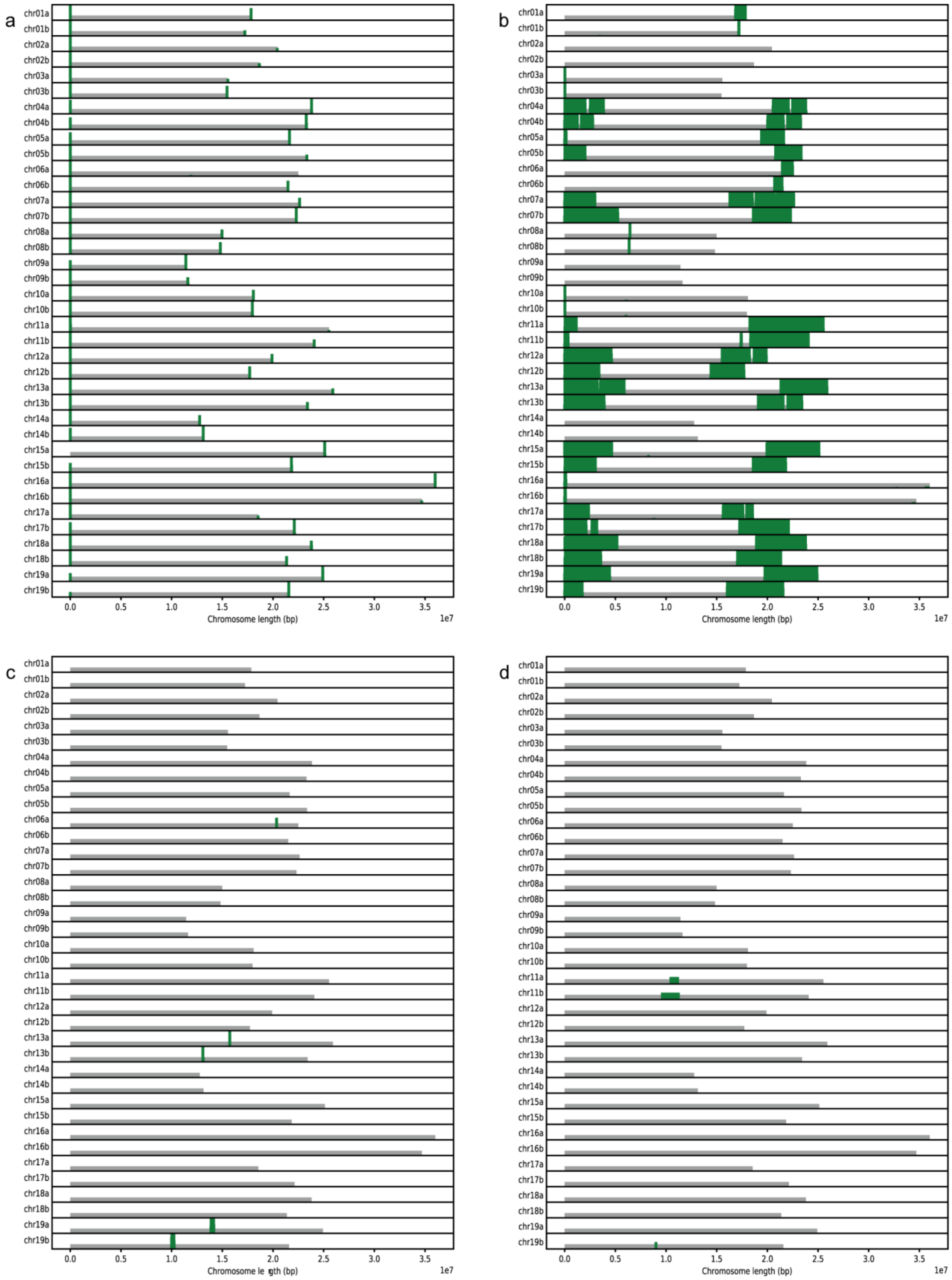

Supplementary Fig. 3. Bar plots of distribution of several characteristic sequences across 38 chromosomes. The green means the characteristic sequences position. (a) Telomeres. (b) Tandem repeat regions. (c) 5S rDNA. (d) 18-5.8-28S rDNA.

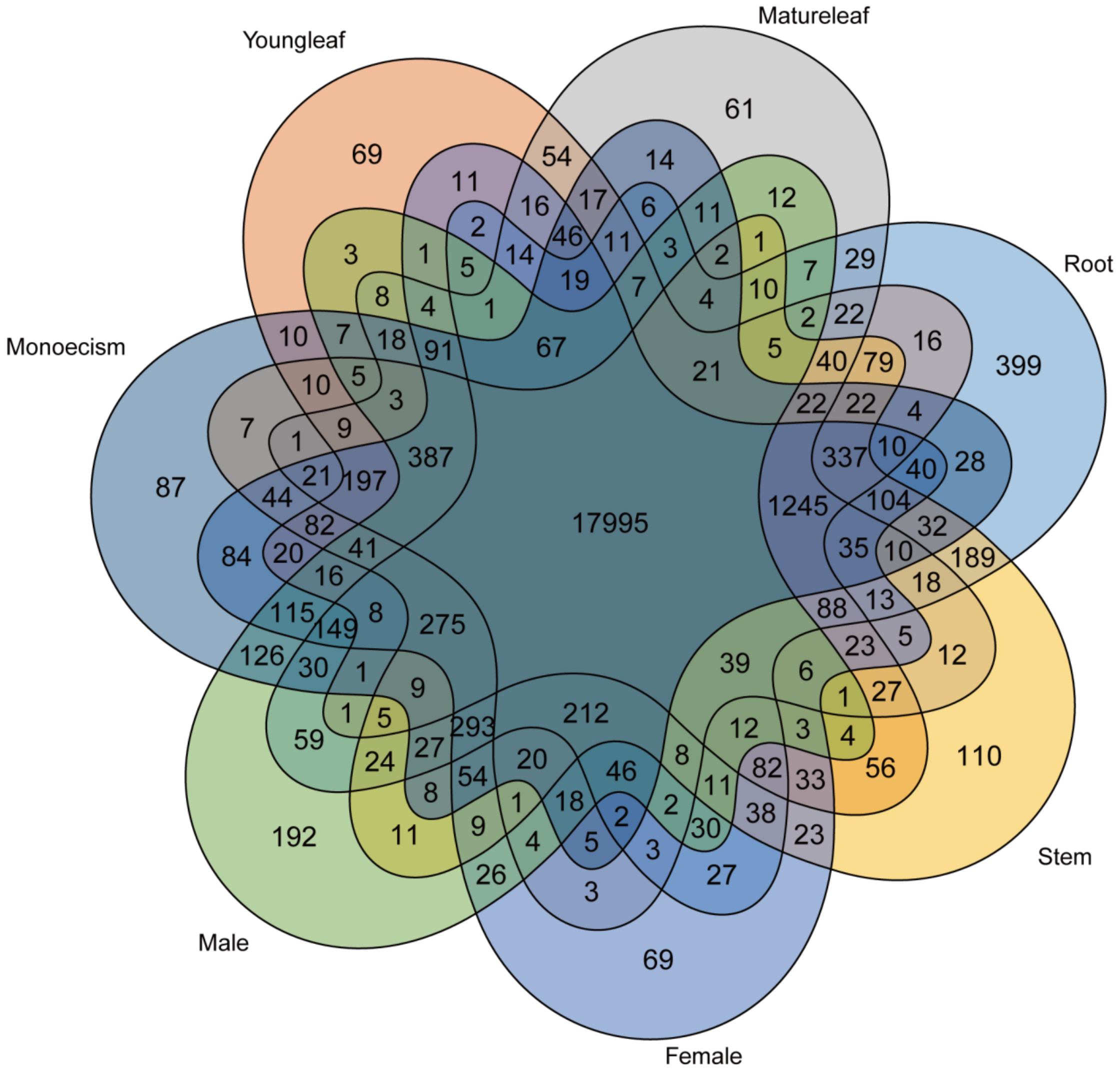

Supplementary Fig. 4. A Venn of specific-organ expression genes. The different colors represent different organs.

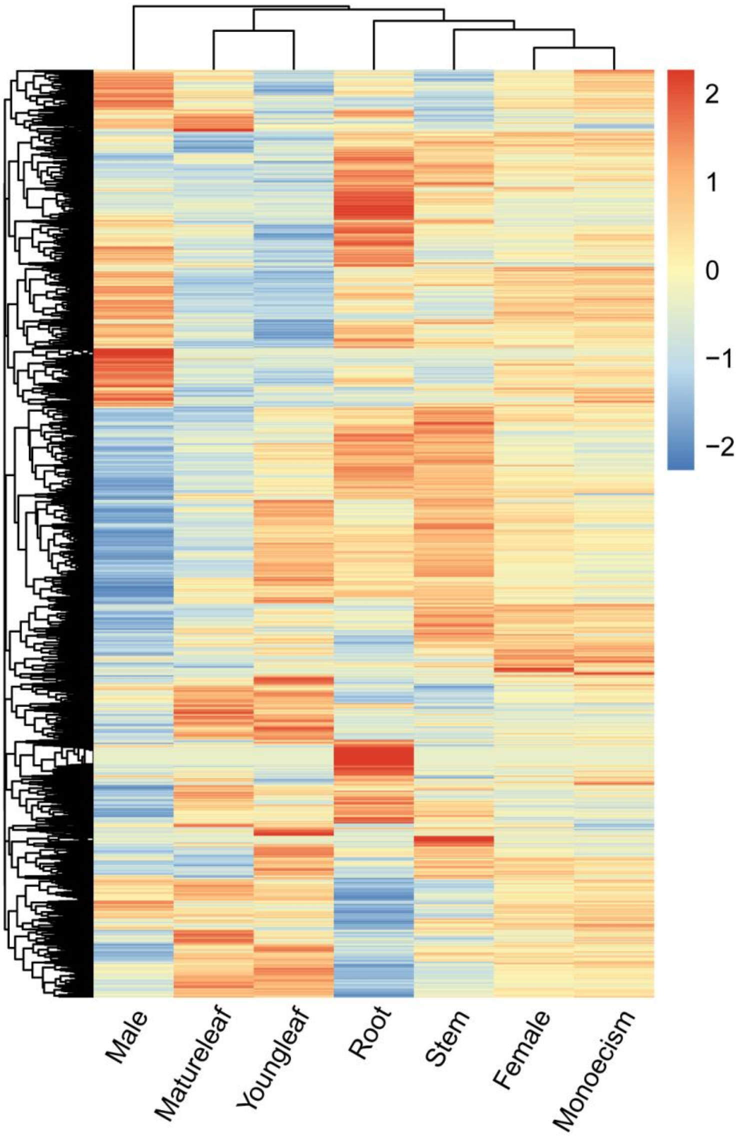

Supplementary Fig. 5. A heatmap of 17,387 DEGs among seven tissues. The expression level was normalized by row.

Supplementary Table 1. Statistics of the long-read HiFi data, Hi-C data and full-length transcripts data.

	Raw reads (M)	Raw bases (Gb)	Clean reads (M)	Clean bases (Gb)	Clean N50 (bp)	Clean L50 (bp)	Clean Q30 (%)	GC content (%)	Max. (bp)	Min. (bp)	Average length (bp)	Coverge
HiFi reads	2.11	38.34	1.78	32.08	17,930	932,517	-	35.38	49,748	44	18,144	100×
Hi-C reads	330.98	**49.65**	323.96	48.59	150	150	91.58	-	150	150	150	126×
Full-length trans reads	17.82	17.45	16.45	16.15	1,194	4,345,931	-	42.92	79,715	88	981	42×

Supplementary Table 2. Transcriptome data of each sample statistics.

Sample ID	Organ	Raw reads(M)	Raw bases(G)	Raw Q30(%)	Clean reads(M)	Clean bases(G)	Clean Q30(%)	GC content(%)	Average length(bp)
S21G0284	female	22	4.2	92.87	21.6	3.1	98.35	45.85	148
S21G0285	young leaf	18.7	5.6	92.42	18.4	2.6	98.20	45.76	147
S21G0286	mature leaf	22.1	5.3	92.59	21.7	3.1	98.27	46.29	148
S21G0287	root	20.7	4.9	92.57	20.3	2.9	98.29	47.62	148
S21G0288	stem	21	6.3	92.63	20.7	3	98.24	45.33	147
S21G0289	root	19.5	5.8	92.19	19.1	2.8	98.17	46.11	148
S21G0290	young leaf	18.5	5.6	91.95	18.2	2.6	98.08	46.33	148
S21G0291	mature leaf	20.2	6.1	92.57	19.8	2.9	98.27	46.45	148
S21G0292	female	19.1	5.7	92.11	18.7	2.7	98.13	45.62	148
S21G0293	stem	22.4	4.4	92.79	21.9	3.2	98.34	45.61	148
S21G0294	root	22.1	4.8	91.96	21.7	3.1	98.08	46.20	147
S21G0295	young leaf	20.6	6.2	92.57	20.2	2.9	98.25	45.70	148
S21G0296	mature leaf	18.2	5.5	92.73	17.9	2.6	98.33	46.27	148
S21G0297	female	16.9	4.2	92.52	16.5	2.4	98.29	45.18	148
S21G0298	stem	19.2	5.8	92.67	18.9	2.7	98.28	45.39	148
S21G0299	male	8.5	2.5	92.47	8.3	1.2	98.28	46.01	147
S21G0300	male	14.8	4.5	92.89	14.7	2.1	98.28	45.84	148
S21G0301	male	20.4	6.1	92.46	20	2.9	98.27	45.90	148
S21G0302	monoecious	20.4	4.8	92.57	20.2	2.9	98.19	45.91	148
S21G0303	monoecious	21.3	5.1	92.84	21.1	3	98.27	45.52	148
S21G0304	monoecious	18.9	5.7	93.00	18.7	2.7	98.29	45.52	148

Supplementary Table 3. Statistics of the chromosomal level assembly S. brachista.

Chr ID	Length (bp)	Contig number	Gap number	Gap length (bp)
chr01a	17,861,335	1	0	0
chr01b	17,228,725	1	0	0
chr02a	20,439,513	1	0	0
chr02b	18,667,281	1	0	0
chr03a	15,550,447	1	0	0
chr03b	15,478,125	1	0	0
chr04a	23,839,391	1	0	0
chr04b	23,302,392	1	0	0
chr05a	21,637,261	1	0	0
chr05b	23,363,187	1	0	0
chr06a	22,503,130	1	0	0
chr06b	21,499,706	1	0	0
chr07a	22,629,168	1	0	0
chr07b	22,312,318	1	0	0
chr08a	14,982,111	1	0	0
chr08b	14,821,491	1	0	0
chr09a	11,424,557	1	0	0
chr09b	11,622,248	1	0	0
chr10a	18,079,726	1	0	0
chr10b	17,983,182	1	0	0
chr11a	25,529,441	1	0	0
chr11b	24,071,074	1	0	0
chr12a	19,913,957	1	0	0
chr12b	17,716,617	1	0	0
chr13a	25,909,808	1	0	0
chr13b	23,420,718	1	0	0
chr14a	12,772,685	1	0	0
chr14b	13,139,249	1	0	0
chr15a	25,121,749	2	1	100
chr15b	21,845,516	2	1	100
chr16a	36,017,666	1	0	0
chr16b	34,689,865	1	0	0
chr17a	18,550,349	1	0	0
chr17b	22,122,785	1	0	0
chr18a	23,806,130	1	0	0
chr18b	21,361,400	1	0	0
chr19a	24,926,306	1	0	0
chr19b	21,577,110	1	0	0

Total length	787,717,719 (99.90%)	1	0	0
Mt	630,081 (0.08%)	1	0	0
Pt	155,612 (0.02%)	1	0	0
tg	0	0	0	0

Note: chr is chromosomes, tg is scattered sequences, Pt and Mt are chloroplasts and mitochondria, respectively.

Supplementary Table 4. Repeat annotations of the S. brachista genome assembly.

Order	Superfamily	Number	Length(bp)	Percent(%)	Mean length(bp)
LTR	Total	211,799	125,865,993	15.96	594.3
	Copia	51,981	48,965,945	6.21	942.0
	Gypsy	53,704	36,536,663	4.63	680.3
	Unknown	106,114	40,363,385	5.12	380.4
Pararetrovirus	-	141	176,777	0.02	1253.7
LINE	-	1,032	1,025,327	0.13	993.5
	Unknown	1,032	1,025,327	0.13	993.5
DNA	Total	429,191	106,164,651	13.46	247.4
	DTA	13,730	5,919,351	0.75	431.1
	DTC	10,156	3,937,264	0.50	387.7
	DTH	6,411	2,889,415	0.37	450.7
	DTM	10,972	3,313,490	0.42	302.0
	DTT	940	239,480	0.03	254.8
	Helitron	386,982	89,865,651	11.40	232.2
MITE	Total	4,625	1,466,742	0.19	317.1
	DTA	3,092	1,189,621	0.15	384.7
	DTC	66	17,561	0.00	266.1
	DTH	165	32,114	0.00	194.6
	DTM	1,296	225,068	0.03	173.7
	DTT	6	2,378	0.00	396.3
Unknown	-	202,592	172,615,028	21.89	852.0
Simple_repeat	-	243,797	11,322,874	1.44	46.4
Low_complexity	-	45,061	2,230,818	0.28	49.5
Total	-	1,138,238	420,868,210	53.38	369.8

Supplementary Table 5. Annotations of the S. brachista genome assembly.

Feature		Number	Min.(bp)	Max.（bp）	Median (bp)	Mean (bp)
Gene	Total	57,169	56	384,610	3,042	4,013.0
	Transcript	90,195	56	17,997	1,945	2,129.5
	CDS	86,264	153	16,350	1,155	1,391.9
	Exon	581,896	3	16,988	154	330.1
	Intron	491,701	4	375,794	186	443.9
Coding gene	Total	53,238	153	384,610	3,256	4,282.2
	Transcript	86,264	153	17,997	1,996	2,209.8
	CDS	86,264	153	16,350	1,155	1,391.9
	Exon	577,816	3	16,988	156	329.9
	Intron	491,558	21	375,794	186	444.1
rRNA	-	1,414	-	-	-	-
tRNA	-	1,301	-	-	-	-
ncRNA	-	1,216	-	-	-	-

Supplementary Table 6. The identification results of the S. brachista allele genes.

Chr	Ref gene	Allele gene	Tandem gene	Paralog gene
Chr01	1,520	1,520	84	161
Chr02	1,731	1,731	15	64
Chr03	1,332	1,332	27	62
Chr04	1,297	1,297	14	42
Chr05	1,565	1,565	13	67
Chr06	1,813	1,813	19	77
Chr07	958	958	22	43
Chr08	1,409	1,409	19	73
Chr09	1,137	1,137	18	58
Chr10	1,566	1,566	25	58
Chr11	906	906	58	89
Chr12	834	834	12	62
Chr13	893	893	39	36
Chr14	1,220	1,220	17	81
Chr15	997	997	36	155
Chr16	2,242	2,242	66	111
Chr17	825	825	72	92
Chr18	825	825	24	41
Chr19	674	674	67	87
Total	23,744	23,744	647	1,459

Supplementary Table 7. The alignment rate of clean data in 21 samples.

Sample ID	Organ	Alignment rate
S21G0284	female	90.16%
S21G0285	young leaf	88.03%
S21G0286	mature leaf	87.99%
S21G0287	root	64.55%
S21G0288	stem	90.20%
S21G0289	root	75.97%
S21G0290	young leaf	87.67%
S21G0291	mature leaf	88.67%
S21G0292	female	89.66%
S21G0293	stem	90.30%
S21G0294	root	80.27%
S21G0295	young leaf	87.08%
S21G0296	mature leaf	88.23%
S21G0297	female	87.55%
S21G0298	stem	90.19%
S21G0299	male	84.50%
S21G0300	male	89.82%
S21G0301	male	90.13%
S21G0302	monoecious	89.65%
S21G0303	monoecious	89.76%
S21G0304	monoecious	88.88%
Average rate		86.63%
